# HIV‐1 proviral landscape characterization varies by pipeline analysis

**DOI:** 10.1002/jia2.25725

**Published:** 2021-07-08

**Authors:** Fernanda A Ferreira, Qianjing He, Stephanie Banning, Olivia Roberts‐Sano, Olivia Wilkins, Daniel R. Kuritzkes, Athe Tsibris

**Affiliations:** ^1^ Virology Program Graduate School of Arts and Sciences Harvard University Cambridge MA USA; ^2^ Division of Infectious Diseases Brigham and Woman’s Hospital Boston MA USA; ^3^ Harvard Medical School Boston MA USA

**Keywords:** proviral landscape, reservoir, HIV‐1, near full‐length genome, provirus characterization, analysis pipeline

## Abstract

**Introduction:**

HIV rebounds after cessation of antiretroviral therapy, representing a barrier to cure. To better understand the virus reservoir, analysis pipelines have been developed that categorize proviral sequences as intact or defective, and further determine the precise nature of the sequence defects that may be present. We investigated the effects that different analysis pipelines had on the characterization of HIV‐1 proviral sequences.

**Methods:**

We used single genome amplification to generate near full‐length (NFL) HIV‐1 proviral DNA sequences, defined as amplicons greater than 8000 base pairs in length, isolated from peripheral blood mononuclear cells (PBMC) of treated suppressed participants with HIV‐1. Amplicons underwent direct next‐generation single genome sequencing and were analysed using four HIV‐1 proviral characterization pipelines. Sequences were characterized as intact or defective; defective sequences were assessed for the number and types of defects present. To confirm and extend our findings, 691 proviruses from the Proviral Sequence Database (PSD) were analysed and the ProSeq‐IT tool of the PSD was used to characterize both the participant and PSD proviruses.

**Results and discussion:**

Virus sequences derived from thirteen ART‐treated virologically suppressed participants with HIV were studied. A total of 693 HIV‐1 proviral sequences were generated, 282 of which were NFL. An average of 53 sequences per participant was analysed. We found that proviruses often harbour multiple sequence defect types (mean 2.7, 95% confidence interval [CI] 2.5, 3.0); the elimination order used by each pipeline affected the percentage of proviruses allotted into each defect category. These differences varied between participants, depending on the number of defect categories present in a given provirus sequence. Pipeline‐specific differences in characterizing the HIV‐1 5′ untranslated region (5′ UTR) led to an overestimation of the number of intact NFL proviral sequences, a finding corroborated in the independent PSD analysis. A comparison of the four published pipelines to ProSeq‐IT found that ProSeq IT was more likely to characterize proviruses as intact.

**Conclusions:**

The choice of pipeline used for HIV‐1 provirus landscape analysis may bias the classification of defective sequences. To improve the comparison of provirus characterizations across research groups, the development of a consensus elimination pipeline should be prioritized.

## 1 Introduction

During HIV‐1 infection, viral DNA integrates into the genomes of CD4^+^ T cells and can persist during antiretroviral therapy [1, 2, 3]. Previous work has investigated what proportion of integrated sequences are intact and therefore may contribute to the replication‐competent HIV‐1 reservoir [4, 5, 6, 7, 8]. The vast majority, greater than 90%, of integrated proviruses are defective and cannot support virus replication [4, 6]. When characterizing intact proviral sequences, the nature of the defective proviruses – whether due to large deletions, hypermutation, inversions, premature stop codons or other categories of defects – is typically also reported. These provirus sequence defects arise through different mechanisms that include HIV reverse transcriptase‐induced mutations, template switching during reverse transcription and APOBEC3‐mediated cytidine deamination that causes guanine‐to‐adenine (G‐to‐A) hypermutation [4, 9, 10].

Current proviral landscape research focuses primarily on the dichotomy between defective versus intact sequences, though “defective” proviruses may still retain transcriptional and translation activity that is relevant to HIV eradication efforts [8, 11]. In general, research groups that characterize provirus sequences use custom in‐house analysis pipelines. Some laboratories categorize amplified DNA sequences manually, whereas others have developed automated, scripted pipelines [5, 7]. While most pipelines include all the classic provirus defect categories (e.g. hypermutation, large deletions, etc.) the order in which defective proviruses are categorized and removed from the pipeline may differ. It is therefore possible that different research groups may arrive at similar conclusions as to the percentage of intact proviruses, whereas the proportion of proviruses assigned into each defect category varies. If true, the direct comparisons of HIV provirus characterizations across research groups may be more difficult and could compromise a deeper, meta‐analytic understanding of the defective nature of the HIV reservoir. Furthermore, recent work has moved beyond a dichotomous classification of intact and defective, focusing on forces that shape the proviral landscape; for example cytotoxic T lymphocytes (CTLs) may preferentially target cells with hypermutated or PSI/MSD defective proviruses [11].

To determine the effect that a particular analysis pipeline may have on defective HIV‐1 proviral characterization, we categorized the proviral HIV‐1 landscapes of 13 HIV‐1 study participants using elimination pipelines from four research groups and explored how the proportion of sequences assigned to each defect category varied as a function of the particular analysis pipeline used. To confirm our findings in this participant cohort and enhance the significance of our conclusions, we performed a similar analysis using sequences retrieved from the Proviral Sequence Database (PSD), a freely available online resource. We found that proviral sequences can typically be assigned into multiple defect categories and the order of elimination of the pipeline matters. Not only does the proportion of sequences within each defect category change with the pipeline used, but differences in how defects are determined by each pipeline has an unexpected effect on the reported percentage of intact HIV‐1 proviral sequences.

## 2 Methods

### 2.1 Participant cohort

Cryopreserved peripheral blood mononuclear cells (PBMC) were obtained from the HIV Eradication and Latency (HEAL) cohort, a longitudinal repository of samples collected from participants with HIV. Peripheral venipuncture samples were collected between 2015 and 2018 and PBMCs were isolated by Ficoll‐Hypaque density gradient centrifugation. All participants gave informed consent prior to enrolment; the HEAL study was approved by the Partners Human Research Committee. Treated virologically suppressed participants with HIV were included in this analysis based on the availability of a requisite number of PBMC.

### 2.2 Proviral amplification and sequencing

Total DNA isolated from participant PBMC samples was subjected to limiting dilution to determine the dilution at which less than 33% of wells were positive for amplified product prior to near full‐genome amplification with Platinum Taq HiFi polymerase (Thermo Fisher Scientific). HIV‐1 proviruses were amplified using previously published PCR conditions and primers [7, 12]. The primers were as follows: first‐round forward primer: 5′‐AAATCTCTAGCAGTGGCGCCCGAACAG‐3′ (HXB2: 623‐649); first‐round reverse primer: 5′‐TGAGGGATCTCTAGTTACCAGAGTC‐3′ (HXB2: 9662‐9686); second‐round forward primer: 5′‐GCGCCCGAACAGGGACYTGAAARCGAAAG‐3′ (HXB2: 638‐666); and second‐round reverse primer: 5′‐GCACTCAAGGCAAGCTTTATTGAGGCTTA‐3′ (HXB2: 9604‐9632). A convenience endpoint of 20 amplified NFL sequences (>8000 base pairs [bp]) per participant was set. This goal was attained for all participants with the exception of HEAL 09, where low levels of total HIV‐1 DNA limited the number of NFL amplicons to nine. PCR amplicons were directly sequenced by next‐generation single‐genome sequencing (NG‐SGS) by the Massachusetts General Hospital Center for Computational and Integrative Biology [MGH CCIB] DNA Core. Briefly, prior to library preparation, the DNA sample was mechanically sheared and Illumina compatible adapters with unique barcodes were ligated onto each sample during library construction. Libraries were pooled in equimolar concentrations for multiplexed sequencing on the Illumina MiSeq platform with 2x150 run parameters, generating approximately 50,000 – 80,000 reads and well greater than 30x coverage of these HIV‐1 amplicons. Sequencing runs routinely generated >90% Q score 30 (Q30) data.

### 2.3 Provirus analysis

Sequenced proviruses were manually analysed using Geneious Prime (www.geneious.com) and classified as either containing a large deletion (<8000 bp in total length), hypermutated, carrying a six nucleotide or longer deletion in the 5′‐untranslated region (UTR), carrying an internal deletion (INDEL), having inversions, having stop codons, carrying a frameshift mutation, carrying a deletion in one of the four stem loops of the packaging signal (PSI) and/or a mutation in the multiple splice donor (MSD) site, or intact. Only sequences with a length greater than 8000 bp were analysed for the other defect categories. Proviruses with APOBEC3G‐mediated hypermutation were identified using the Los Alamos National Laboratory (LANL) HIV Sequence Database Hypermut algorithm [13]. The LANL HIV Sequence Database Gene Cutter tool was used to identify stop codons and frameshift mutations [14]. INDELs were identified using the LANL HIV Sequence Database HIVAlign tool and defined as an equal or greater than 2% difference in the sequence of each HIV gene [15]. 5′‐UTR deletions (set at 6 or more deletions), PSI deletions and MSD mutations were identified by aligning all the sequences to the HIV‐1 HXB2 reference sequence (Geneious Prime 2019.1.1) and checked for deletions and mutations [16]. Inversions were identified by aligning each proviral sequence to HXB2 and comparing them using the Dotplot viewer in Geneious Prime [16]. Proviral sequences that lacked any of these defects were labelled as intact.

### 2.4 Proviral sequence database and ProSeq‐IT

Proviral sequences from virologically suppressed HIV‐1 participants on ART were downloaded from the Proviral Sequence Database (https://psd.cancer.gov) (PSD) maintained by the National Cancer Institute (NCI) [17]. A total of 691 proviruses were retrieved; 129 were NFL sequences. Intact and defective sequences were manually determined and the proviral landscape was characterized with the four pipelines. Additionally, the Proviral Sequence Annotation and Intactness Test (ProSeq‐IT) of the NCI PSD independently analysed the 282 NFL sequences from the 13 HEAL participants and the 691 NFL sequences from PSD [18].

## 3 Results

### 3.1 Participant characteristics

HIV‐1 proviral sequences were amplified from PBMC DNA isolated from thirteen participants. The participants’ characteristics are shown in Table 1. These participants were almost exclusively male with a mean CD4 count of 807 cells/mm^3^ (SD = 393). Participants were receiving antiretroviral therapy, comprised of at least three active drugs, for a minimum of 6 months; all had plasma HIV‐1 RNA levels that were below the limit of detection of a commercially available virus load assay.

**Table 1:** Characteristics of study subjects

Patient ID no.	Sex	Age	CD4^+^ T cell count (cells/mm^3^)	Therapeutic regimen	Months with viral load < 50 copies/mL
01	M	54	606	3TC, ABC, EFV	24
04	M	37	773	DRV/cobi, FTC TAF	>80
09	F	32	1713	EVG/cobi, FTC, TDF	44
16	M	56	267	ETR, FTC, RAL, TDF	10
19	M	54	416	DRV/r, DTG, FTC, MVC, TAF	6
24	M	55	691	EVG/cobi, FTC, TAF	12
25	M	31	984	EVG/cobi, FTC, TAF	12
30	M	61	1005	EFV, FTC, TDF	>27
38	M	52	695	DTG, FTC, TAF	>27
44	M	58	448	DTG, FTC, TAF	16
50	M	65	899	DTG, FTC, RPV, TAF	42
57	M	58	1346	EVG/cobi, FTC, TAF	17
58	M	45	647	DRV/cobi, FTC, TAF	29

3TC, lamivudine; ABC, abacavir; BIC, bictegravir; DRV/cobi, cobicistat boosted darunavir; DRV/r, ritonavir boosted darunavir; DTG, dolutegravir; EFV, efavirenz; ETR, etravirine; EVG/cobi, cobicistat boosted elvitegravir; FTC, emtricitabine; MVC, maraviroc; RAL, raltegravir; RPV, rilpivirine; TAF, tenofovir alafenamide; TDF, tenofovir disoproxil fumarate.

### 3.2 Characterization of HIV‐1 proviral defects in the HIV‐1 participants

**Figure 1:**
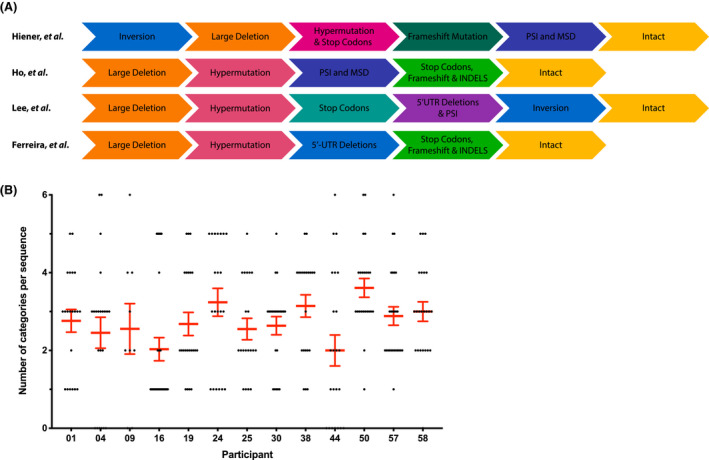
Analysis pipeline schema and sequence categorization summary. **(A)** Comparison of the ordering processes of sequence elimination used by four research groups to identify NFL intact HIV‐1 proviruses. Adapted from Ref. 1, 2 and 8. **(B)** Comparison of the number of categories assigned to each amplified sequence for every study participant. For amplified provirus sequences >8000 bp there are six defect categories they can be assigned to hypermutation, 5′‐UTR deletion, PSI deletion and/or MSD mutation, INDEL, inversion and stop codon and/or frameshift. Each point represents a single amplified sequence and the mean and SEM are shown in red.

We assessed the proviral characterization pipelines from three research groups, as well as our own, based on their published descriptions and personal communication (Figure 1) [4, 5, 7]. We used the same definition of a given defect category across all pipelines, unless noted, but acknowledge that our stringency may differ from that originally used by each research group. For instance Hiener *et al*. define all proviral sequences below 8400 bp as containing a large deletion, whereas we used an 8000 bp cut off.

To understand how different analysis pipelines affect sequence identification and categorization, we used each of the pipelines to analyse 693 HIV‐1 proviral sequences isolated from the thirteen participants. An average of 53 sequences were analysed per participant; 12 of 13 participants had at least 20 NFL sequences. Of the 282 NFL sequences, 229 (81.2%) carried more than one type of defect and the distribution of the number of defect categories per provirus sequence varied across participants (Figure 1b). On average, a defective sequence could be assigned to 2.7 (95% CI 2.5, 3.0) defect categories (median 3 categories). For some participants, for example HEAL 04, 09 and 58, all defective sequences contained two or more defect categories. For HEAL 16 on the other hand, the majority (63%) of defective NFL sequences were assigned to a single category, with the remaining sequences assigned to two, four or five categories.

Hypermutation leads to stop codons, which is why some pipelines combine the two categories or characterize only hypermutation [5, 7]. However, for certain participants (e.g. HEALs 01, 16, 25, 30 and 58), we observed instances where stop codons occurred without hypermutation. In HEALs 16, 25 and 30, for example none of the NFL sequences with stop codons displayed G‐to‐A hypermutation. For HEAL 01 and 58, two and four NFL sequences, respectively, had stop codons not related to hypermutation.

**Figure 2:**
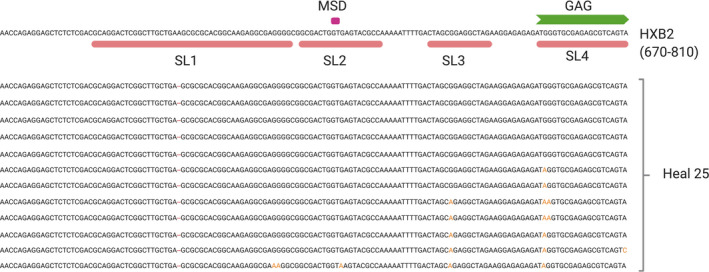
Defects in PSI impact the determination of provirus intactness. Defining a provirus as defective based on deletions in the stem loops of the PSI increases the number of proviral sequences deemed defective, when compared to looking for 6 or more deletions in the 5′‐UTR. All 12 HEAL 25 provirus sequences shown contain a single deletion in SL1 and are considered defective when using the more stringent PSI deletion definition but are not considered defective using the more general 5′‐UTR defect category. Note that the nucleotide sequence proximal to the dimerization motif may differ across virus isolates; intactness should not imply replication competence, and vice versa. A subset of proviral sequences from Heal 25 is shown here with the HIV‐1 reference sequence, HXB2. The four stem loops of the HIV‐1 PSI are shown in salmon, the MSD site is shown in magenta and the start of GAG in green.

Among the 13 study participants, there were only two instances where we observed a perfect concordance between sequences containing six or more nucleotide deletions in the 5′‐UTR and those containing a deletion in the PSI stem loops and/or a mutation at the MSD site: HEAL participants 01 and 38. For the other eleven participants, deletions in PSI and/or mutations in MSD were not captured by the more general definition of a defective 5′ UTR; the more precise the definition of the defect category, the greater the number of sequences that are considered defective (Figure 2). This is illustrated by proviral sequences obtained from participant HEAL 25, where the number of NFL sequences with six or more deletions in the 5′‐UTR is one, but that number jumps to 12 when assessing for PSI deletions, in this case, due to a single deletion in the conserved hairpin loop of stem loop 1 (SL1) immediately proximal to the palindromic dimerization sequence. Adjusting this category to include deletions in the PSI increases the number of defective sequences in this category for the majority of participants: 01 (0 to 8), 04 (9 to 10), 24 (1 to 7), 25 (1 to 9), 50 (8 to 13) and 58 (0 to 13). Furthermore, not including MSD mutations, as is the case for two pipelines, also inflates the number of intact proviruses in five HEAL participants: 16 (1 to 2), 24 (0 to 6), 25 (0 to 4), 38 (0 to 1), 44 (7 to 11) and 50 (0 to 2).

### 3.3 The effect of pipeline on defective sequence proportions

**Figure 3:**
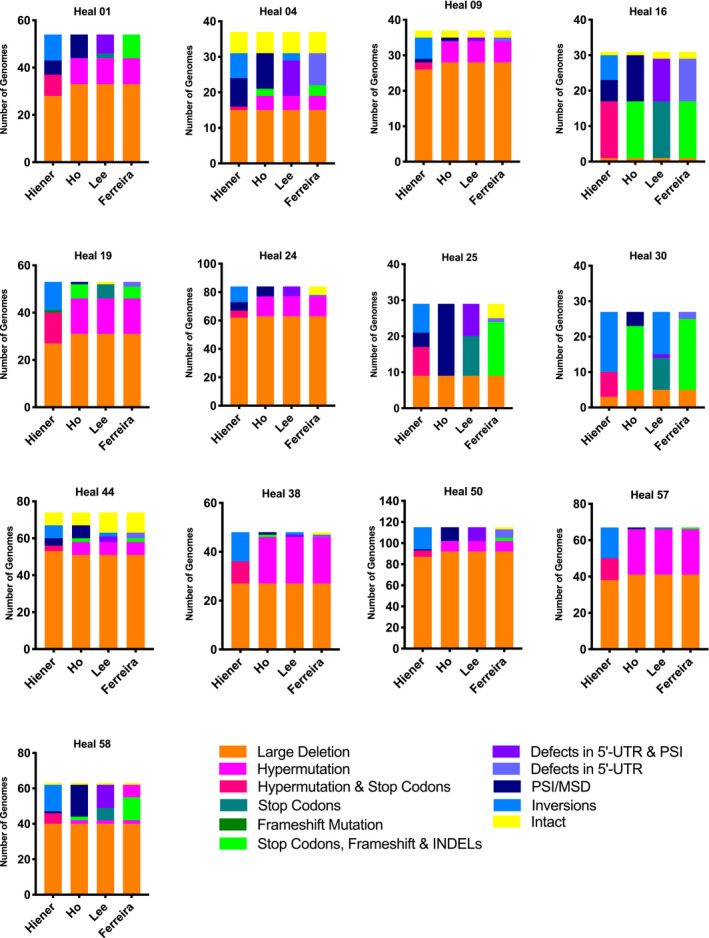
Characterization pipelines and their effect on the HIV‐1 proviral landscape. The number of genomes allotted into each category within the total pool of sequenced proviruses by each pipeline investigated. The four pipelines are, from left to right, Hiener *et al*., 2018; Ho *et al*., 2013; Lee *et al*., 2017; and Ferreira, our own pipeline.

**Figure 4:**
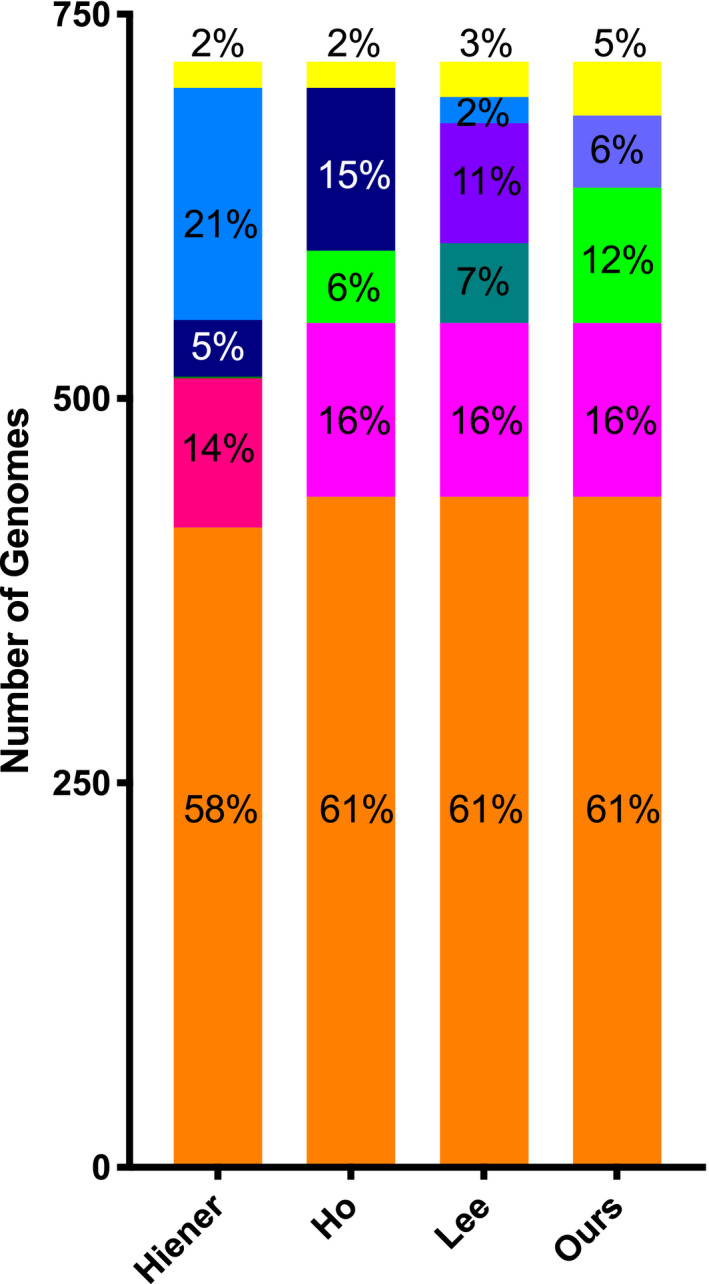
Comparison of the cumulative landscape for the study cohort generated using the four pipelines. The number of genomes in each defect category for all 693 proviral amplicons according to the four pipelines analysed, showing the percentage of genomes in each category. Orange, large deletion; magenta, hypermutation; pink, hypermutations and stop codons; dark blue, PSI/MSD defects; light blue, inversions; dark green, stop codons; green, stop codons, frameshifts, and indels; light purple, defects in 5′ UTR; purple, defects in 5′ UTR and PSI; yellow, intact.

Given that 81.2% (229 of 282 sequences) of NFL proviral sequences contain two or more types of defect, the order of elimination used by a pipeline, as well as the inclusion or exclusion of certain categories, may influence the final proviral landscape. To evaluate this, we analysed, on a per‐participant basis, how the percentage of sequences assigned to each category changes as a function of the pipeline used (Figure 3). We also examined the effect of the pipeline on the cumulative proviral landscape for all 13 participants (Figure 4).

How certain pipelines combine HIV‐1 sequence defect categories has an influence on the final landscape. “Stop Codons” are their own category in Lee *et al*. but are combined with “Hypermutation” in Hiener *et al*. and with “Frameshift Mutations” and “INDELs” in Ho *et al*. HEAL 16 is the only participant where this has no effect on the number of genomes in this category: there are always 16 sequences assigned independently of whether “Stop Codons” are combined with any other type of defect. HEAL 16 is unique in this respect because approximately 63% of HEAL 16 proviruses have a single defect. For the other 12 participants, however, the combination of “Stop Codons” with other defects leads to slight discrepancies in the percentage of sequences in each category, as exemplified by HEAL 38 where the number of sequences in a “Stop Codon” category varies from 9 to 0 depending on the pipeline.

The elimination order of the pipeline also influences the final percentage of sequences in each category and this is particularly evident in the case of hypermutation. For instance for HEAL 38, 19 provirus sequences are hypermutated according to three of the pipelines, but this number falls to 9 when sequences are checked for inversions before hypermutations. The same happens for HEALs 01 (11 to 9), 04 (4 to 1), 09 (6 to 2), 19 (15 to 13), 24 (14 to 5), 44 (7 to 3), 50 (10 to 6) and 57 (25 to 12).

An “Inversions” defect category is included in two pipelines, though the order in which it appears is different in each: inversions are the first defect category in the Hiener *et al*., pipeline, but the last category in Lee *et al*. Unsurprisingly, while the Hiener *et al*. pipeline leads to 21% of all proviruses categorized as having inversions in the cumulative proviral landscape of all 13 participants, this percentage falls to 2.4% with the Lee *et al*. pipeline.

Across all pipelines, the “Large Deletion” category has the similar total number of genomes: 436 for all pipelines, with the exception of Hiener *et al*. where there are 416 genomes. The percentage of intact and hypermutated genomes is also similar across the four pipelines (between 2.4% and 4.9% for intact and 13.5 and 15.7% for hypermutated) and in line with what has been previously shown for HIV‐1 [1, 2, 4, 8]. For the other categories, however, the number of genomes in each category differs substantially. Looking generally at defects in the 5′‐UTR, the number of genomes in this region varies from 37 (5.1%) to 106 (14.8%) depending on the specificity of the defect category and when it appears in the pipeline. The same is seen with defect categories involving “Stop Codons,” which can represent anywhere from 6.5 to 12.2% of the proviral landscape.

### 3.4 Characterization of HIV‐1 proviral defects in the proviral sequence database

**Figure 5:**
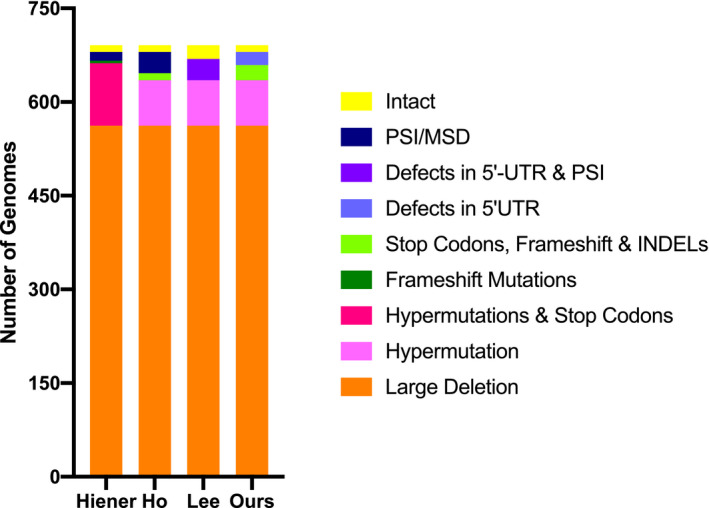
The effect of the characterization pipelines on the proviral landscape of the 691 HIV‐1 sequences retrieved from the PSD Database. The four pipelines are, from left to right, Hiener *et al*., 2018; Lee *et al*., 2017; Ho *et al*., 2013; and our own pipeline.

To assess whether the choice of characterization pipeline continues to have an impact on a collection of provirus sequences coming from a number of research groups, where both experimental and sequence analyses differences may lead to inter‐laboratory variability, we retrieved provirus sequences from the Proviral Sequence Database (PSD). We selected proviruses from HIV‐1 participants on ART with undetectable plasma viral loads. A total of 691 provirus sequences were identified, of which 129 were NFL. We then used the three published pipelines, as well as our own, to characterize the proviral landscape of these 691 proviruses (Figure 5). All pipelines generated similar percentages of sequences with large deletions (81.2%); none of the NFL proviruses had inversions. The number of proviruses assigned to a defect category varied by the stringency of the category, the order in which it appears in the pipeline, and how different types of defects were grouped into a single category. Using the PSD dataset, we recapitulated our finding that the analysis pipeline may affect the percentage of intact sequences, varying two‐fold across the pipelines we studied.

The PSD provides a tool (ProSeqIT) that annotates sequences and infers intactness. ProSeq‐IT identified 39 (5.6%) of the 691 PSD provirus sequences as intact, a higher proportion of intact sequences than with the four pipelines we tested. To determine how the ProSeqIT tool characterized our NFL HEAL cohort‐derived provirus sequence set, we submitted the 282 NFL sequences to ProSeq‐IT, which called 6.0% of sequences as intact, again a higher proportion than was determined in the four analysis pipelines. A more detailed analysis identified that this difference in the proportion of sequences called intact was driven primarily by differences in calling PSI deletions, but there were also differences in calling inversions, stop codons/frameshifts and in one case hypermutation.

## 4 Discussion

In this study, our analysis of four proviral landscape characterization pipelines suggests that the choice of pipeline influences both the proviral landscape of an individual participant as well as the cumulative proviral landscape of a study cohort. The pipeline’s elimination order can influence the number of genomes allotted into each defect category, as well as the number of sequences deemed intact. This occurs because the majority of amplified provirus sequences are defective in more than one way. The exact category definitions used also influence the final proviral landscape for each participant and, in general, the more specific a defect category, the fewer sequences that are deemed intact by the end of the pipeline.

While the final step in all pipelines defines which proviral sequences are intact, there is marked heterogeneity in the preceding steps. Large deletions and hypermutated sequences are generally identified early in the process. Inversions are categorized either first, in the penultimate step, or are not explicitly categorized. The category for defects in the 5′‐untranslated region (UTR) is defined as six or more deletions anywhere along its length or more specifically as deletions in the PSI packaging signal and, in some pipelines, mutations at the major splice donor (MSD) site. An evaluation for frameshift mutations, nonsense mutations, stop codons and insertions/deletions (indels) occur in all pathways but there are differences across pipelines. The order of these steps varies, as do the permutations on how they may be performed in one combined sequence step. All pipelines examine for the presence of hypermutated sequences, though when it is assessed in the pipeline varies. We found that the further downstream in the pipeline the “Hypermutation” category appears, the fewer sequences are categorized as hypermutated. Given the different pipelines and that the majority of NFL sequences carry at least two types of defects, it is perhaps less surprising that the cumulative proviral landscape varies according to the pipeline used.

We observed an association between certain defect categories that varied across participants. By looking only at hypermutation, sequences with stop codons that were not the result of APOBEC‐mediated G‐to‐A mutations may be considered intact or placed in a different defect category. Alternatively, combining hypermutation and stop codons into a single category of defects may inflate the percentage of the proviral landscape that is deemed hypermutated. We further observed an association between 5′‐UTR deletions, PSI deletions and MSD mutations. Considering only 5′‐UTR deletions led our pipeline to erroneously overestimate the number of intact NFL sequences. This demonstrates that the specific pipeline used can influence not just the number of provirus sequences in each category, but also the number of sequences considered intact. We found that a more stringent approach to evaluate the 5′‐UTR, using both PSI deletions and MSD mutations, led to fewer intact sequences. Work exploring how CTLs shape the proviral landscape have looked at PSI/MSD defects; we found that the percentage of genomes in the 5′‐UTR defect group varied from 5% to 15% depending on the pipeline, something to be aware of when looking at landscape results across research groups.

Our study has limitations. We did not assess the replication competence of the NFL proviral sequences deemed intact by the four pipelines. A NFL provirus sequence that is intact via the pipelines may prove replication competent in an in vitro experiment. Conversely, the relationship between smaller deletions in sequences labelled defective, for example the SL1 mutations we identified in proviruses and exemplified in HEAL 25 (Figure 2) and replication competence was not tested.

We acknowledge that research groups may have different defect definitions – or may have since updated their definitions to increase stringency. Inter‐laboratory variability may arise from differences in sequence analysis pipelines, alignment strategies, and/or sample processing and sequencing approaches. The choice of primers, polymerase and Poisson frequency target in limiting dilution could influence the final proviral landscape for a participant or a cohort. Intra‐participant and inter‐participant sequence diversity may impact these analyses and could be explored in future studies. We tried to control for inter‐laboratory differences by running the pipelines on 693 proviruses amplified using the exact same protocol and approach. Unlike our HEAL cohort, the PSD sequences come from a repository of nearly 5000 proviruses, submitted by various research groups and, potentially, impacted by inter‐laboratory variability. We concede, a priori, that the best choice of pipeline for provirus landscape analysis will depend on the precise research question being asked and that pipelines provide a measure of inferred intactness that should be assessed for replication competence experimentally.

## 5 Conclusions

Our results suggest that the field may benefit from a consensus analysis pipeline. We believe a best practice approach starts with eliminating large deletions (retaining sequences >8000 nucleotides), followed by hypermutations, then a category combining deletions in the PSI and mutation in the MSD, followed by a second combined category of internal deletions, stop codons and frameshift mutations in the HIV ORFs and finally inversions. To improve transparency and mitigate bias, research groups should consider analysing NFL proviral sequences for all defect categories, allowing for the identification of provirus sequences that carry multiple types of defects and generating information about how defect categories overlap. While this approach may lead to more complex results, it will provide a richer illustration of the proviral landscape. This may be especially important if a proviral sequence with multiple defects expands clonally. If such proviruses are only analysed for a single defect, the presence of additional accompanying defects may be underestimated or missed.

## Competing interests

The HEAL repository is supported in part through research funding from Merck & Co. AT receives research funding and financial remuneration from Merck and Co and Gilead Sciences, Inc.

## Author’ contributions

F.A.F. and A.T. conceptualized and designed the study, analysed the data and wrote the manuscript. F.A.F. performed the research and created the data visualizations. D.R.K reviewed and edited the manuscript. Q.H,. S.B. and O.R.S. processed participant samples and isolated PBMC for subsequent analyses. O.W. recruited the participants into the HEAL cohort. D.R.K. and A.T. supervised the work; A.T. was responsible for funding acquisition.
